# Pathogen Sensing Pathways in Human Embryonic Stem Cell Derived-Endothelial Cells: Role of NOD1 Receptors

**DOI:** 10.1371/journal.pone.0091119

**Published:** 2014-04-01

**Authors:** Daniel M. Reed, Gabor Foldes, Timothy Gatheral, Koralia E. Paschalaki, Zsuzsanna Lendvai, Zsolt Bagyura, Tamas Nemeth, Judit Skopal, Bela Merkely, Aurica G. Telcian, Leila Gogsadze, Michael R. Edwards, Peter J. Gough, John Bertin, Sebastian L. Johnston, Sian E. Harding, Jane A. Mitchell

**Affiliations:** 1 Department of Cardiothoracic Pharmacology, National Heart and Lung Institute, Imperial College London, London, United Kingdom; 2 Department of Cardiac Pharmacology, National Heart and Lung Institute, Imperial Centre for Translational and Experimental Medicine, Imperial College London, London, United Kingdom; 3 Heart and Vascular Center, Semmelweis University, Budapest, Hungary; 4 Airway Disease Section, National Heart and Lung Institute, Imperial College London, London, United Kingdom; 5 MRC and Asthma UK Centre in Allergic Mechanisms of Asthma and Centre for Respiratory Infection, National Heart and Lung Institute, Imperial College London, London, United Kingdom; 6 Pattern Recognition Receptor Discovery Performance Unit, Immuno-Inflammation Therapeutic Area, GlaxoSmithKline, Philadelphia, Pennsylvania, United States of America; McMaster University, Canada

## Abstract

Human embryonic stem cell-derived endothelial cells (hESC-EC), as well as other stem cell derived endothelial cells, have a range of applications in cardiovascular research and disease treatment. Endothelial cells sense Gram-negative bacteria via the pattern recognition receptors (PRR) Toll-like receptor (TLR)-4 and nucleotide-binding oligomerisation domain-containing protein (NOD)-1. These pathways are important in terms of sensing infection, but TLR4 is also associated with vascular inflammation and atherosclerosis. Here, we have compared TLR4 and NOD1 responses in hESC-EC with those of endothelial cells derived from other stem cells and with human umbilical vein endothelial cells (HUVEC). HUVEC, endothelial cells derived from blood progenitors (blood outgrowth endothelial cells; BOEC), and from induced pluripotent stem cells all displayed both a TLR4 and NOD1 response. However, hESC-EC had no TLR4 function, but did have functional NOD1 receptors. *In vivo* conditioning in nude rats did not confer TLR4 expression in hESC-EC. Despite having no TLR4 function, hESC-EC sensed Gram-negative bacteria, a response that was found to be mediated by NOD1 and the associated RIP2 signalling pathways. Thus, hESC-EC are TLR4 deficient but respond to bacteria via NOD1. This data suggests that hESC-EC may be protected from unwanted TLR4-mediated vascular inflammation, thus offering a potential therapeutic advantage.

## Introduction

Endothelial cells line the luminal surface of blood vessels and provide a physical and metabolic barrier between the vessel and the circulation, and are essential for cardiovascular homeostasis. In health, endothelial cells release vasoactive hormones including prostacyclin and nitric oxide, which regulate smooth muscle and platelet function [1]. Endothelial cells are also a key cell type in innate immunity, and express pattern recognition receptors (PRRs), including Toll like receptors (TLRs) and nucleotide-binding oligomerization domain-containing protein (NOD) receptors [2]–[4]. Gram-negative bacteria are sensed by two key PRRs, TLR4, which recognises lipopolysaccharide (LPS), and NOD1, which recognises moieties in peptidoglycan. Activation of endothelial cells by pathogens is an early event in innate immunity, resulting in the expression of adhesion receptors and the release of chemokines [5], [6]. This allows for immune cells to be recruited to an area of infection, and for subsequent pathogen killing, removal and resolution. However, PRRs on endothelial cells, including TLR4 and TLR2, have also been associated with vascular inflammation and cardiovascular disease, such as atherosclerosis [7]–[9].

The therapeutic potential of stem cell-derived endothelial cells is increasingly recognised. As such, endothelial cells derived from stem cells are currently being investigated as cell therapies for a number of conditions, including cardiovascular disease [10]. The most common sources of stem cells that can be differentiated to endothelial cells include embryonic stem cells, induced pluripotent stem cells and adult progenitor stem cells; each with benefits and limitations. Understanding how stem cell-derived endothelial cells function at both the cardiovascular and immune level will be essential in the arena of cell therapy and organ regeneration, where new vessel and vascular network construction underlies the basis of clinical benefit.

We have previously shown that endothelial cells derived from human embryonic stem cells (hESC-EC) express an immature immune phenotype, with no discernible TLR4 function [11]. We have speculated that this may provide an advantage since TLR4 on endothelial cells is directly linked to atherosclerosis [10], [11]. However, lack of TLR4 could result in endothelial cells not being able to sense pathogens, and render tissue/organs immune-suppressed and thereby susceptible to infection with Gram-negative bacteria. In the current study we have confirmed our previous work that hESC-EC do not express functional TLR4 responses. For the first time, we have compared TLR4 and NOD1 functions in endothelial cells derived from three key stem cell sources; embryonic stem cells, adult progenitors (blood outgrowth endothelial cells; BOEC) and induced pluripotent stem cell derived endothelial cells (iPSC-EC). We also extend our previous work by showing that hESC-EC remain devoid of TLR4 after a period of *in vivo* ‘conditioning’ in nude rats. We have gone on to investigate the functionality of NOD1 receptors in hESC-EC and whether, through NOD1 signalling, hESC-EC can sense live Gram-negative bacteria.

## Materials and Methods

### Media and Solutions

Lonza-EGM2 media was prepared by addition of Lonza-EGM2 SingleQuot supplements and growth factors to Lonza-EBM2 basal medium (Lonza, Belgium). Information of the concentrations of additions in ‘SingleQuot supplements and growth factors’ are not available, however, supplier's information states the following are included; human epidermal growth factor (hEGF), gentamicin-amphotericin-B 100, R3- insulin growth factor (IGF)-1, ascorbic acid, vascular endothelial cell growth factor (VEGF), human fibroblast growth factor (hFGF)-B, heparin, hydrocortisone. The media was prepared according to the supplier's instructions except that the recommended 2% foetal bovine serum (FBS) supplied with the kit was replaced and increased to 10% using FBS from Hyclone (HYC-001-330Y, Thermo Scientific, Massachusetts, USA). Type-1 rat tail collagen solution (#35423, Becton Dickinson, New Jersey, USA) was prepared in 0.02N glacial acetic acid, according to manufacturer's instructions, at a concentration of 50 µg/ml and used to pre-coat surfaces used for blood outgrowth endothelial cell (BOEC) isolation and maintenance. Coating was achieved by adding 5.2 µg/cm^2^ collagen solution and incubating at 37°C, 5% CO2 for 1 hour prior to washing three times with PBS.

### hESC-EC

Experiments were carried out using the H7 hESC line provided under collaboration agreement with the Geron Corporation (Menlo Park, CA, USA) and with permission of the UK Stem Cell Bank. All ethical approvals had been acquired. hESC were maintained in their undifferentiated state as described previously [12] and as instructed by Geron. Briefly, hESC were grown on Matrigel (BD Biosciences) coated 6-well plates in mouse embryonic fibroblast conditioned medium supplemented with 8 ng/ml basic fibroblast growth factor.

Differentiation of hESC into hESC-EC was carried out as described previously [11]. Briefly, cells were dissociated into clumps and plated on ultra-low attachment plates (Nunc, Denmark) with Lonza-EGM2 to allow formation of embryoid bodies. After 4 days embryoid bodies were re-plated on 1% gelatinized (1% gelatin) (Sigma-Aldrich, St Louis, USA) 6-well plates in Lonza-EGM2. After 13 days cells were stained for CD31 using an Alexa Fluor 488 fluorescence dye labelled anti-CD31 antibody (BD Biosciences, Oxford, UK). Cells were sorted using a FACS Aria II cell sorter (BD Biosciences, Oxford, UK) and expanded in Lonza-EGM2 medium for further use.

### Blood outgrowth endothelial cells (BOEC)

BOEC were isolated as published elsewhere [13]–[17] with minor modifications. Briefly, blood (48 ml) was collected into tubes with Ficoll [18], from healthy volunteers aged 24–45 and centrifuged at 1600 RCF for 30 minutes at room temperature with maximum acceleration and braking rates to obtain PBMCs. Tubes were then inverted 8 times prior to centrifugation, and after centrifugation to allow mixing of the buffy coat and plasma/serum fraction. Contents were then carefully pooled into a 50 ml falcon tube and 10% FBS/PBS added to give a final volume of 50 ml. Cells were then centrifuged at 520 RCF for 10 minutes with maximal acceleration and intermediate braking. The supernatant was discarded and pellets resuspended in 10 ml of 10% FBS/PBS solution. This process was repeated a further two times giving three washes in total. Prior to the final centrifugation 10 µl of cell suspension was added to a haemocytometer for counting. After the final wash cells were subsequently resuspended in an appropriate amount of Lonza-EGM2 with 10% FBS and distributed across collagen pre-coated wells of a 6-well plate (Nunc, Denmark) at a density of 3×10^7^ cells/well. Plates were incubated at 37°C, 5% CO_2_. After 24 hours media was carefully removed, cells were washed with Lonza-EGM2 10% FBS and 4 ml of fresh Lonza-EGM2 10% FBS added to each well. This process was repeated every 48 hours for 4 days then every 24 hours until day 7. After day 7 media was replaced every other day without washing until colonies appeared. Colonies of endothelial cells typically emerged between days 7–20. Once colonies emerged they were allowed to expand for not more than 3–5 days. Colonies were removed by trypsin (TrypLE 1×) digest using 2 ml trypsin/well. Trypsin was neutralised with 4 ml Lonza-EGM2 10%FBS and the 6 ml cell/trypsin mix collected in a 50 ml falcon tube and centrifuged at 190 RCF for 5 minutes at room temperature with maximal acceleration and intermediate break settings. Cells were then plated on expanded and maintained on T25 and T75 culture flasks (Nunc, Denmark) pre-treated with collagen as described above.

### Human umbilical vein endothelial cells (HUVEC)

HUVEC were a gift from Caroline Wheeler-Jones (Royal Veterinary College, London), and were isolated as described previously [19]. Cells were maintained in Lonza-EGM2 medium. Cells were at passage 2 on arrival and used for experiments between passage 2–8. HUVEC were grown on gelatinized (1% gelatin) (Sigma-Aldrich, St Louis, USA) T75 flasks.

### Induced pluripotent stem cell derived-endothelial cells (iPSC-EC)

IPSC-EC used in this study were purchased from Cellular Dynamic International (Madison, USA). Cells were maintained in Lonza-EGM2 on fibronectin (Invitrogen, California, USA) coated T75 flasks according to manufacturer's instructions.

### Treatment protocols

Cells were plated on 1% gelatinised 96-well plates (Nunc, Denmark) and grown to confluence. Cells were seeded at a density of 7,000cells/well and confluence was defined as 80–100% coverage. Time to reach confluence was approximately 48 hours. Cells were then treated with media alone or media +/− LPS (0.1–1 µg/ml) (Invivogen, California, USA), C12-iE-DAP [20] (Lauroyl-c-D-Glu-mDAP) (1–10 µg/ml) (Invivogen, California, USA) or IL-1β (0.1–1 ng/ml) (R & D systems, Abingdon, UK) for 1 or 24 hours. Where responses of cells were compared directly, different endothelial cell types were plated in the same media and treated under identical conditions. The RIP2 inhibitor GSK'214 and the NOD1 inhibitor GSK'217 were provided by GlaxoSmithKline (Philadelphia, USA). Precise structural details for GSK2576214A (GSK'214) and GSK1219217A (GSK'217) were not available to us at this time. Cells were incubated with inhibitors for 30 minutes prior to addition of agonists. Drugs were dissolved initially in dimethyl sulphoxide (except for LPS which were dissolved in PBS) to prepare stock solution. Further dilutions were made in Lonza-EGM2 with 10% FBS.

### siRNA knockdown of NOD1

For NOD1 siRNA knockdown protocols, targeting NOD1 siRNA (Hs_CARD4_1 Flexitube siRNA (NM_006092); Qiagen, Crawley, UK) was used according to manufacturer's instructions. Cells were plated 24 hours before transfection. Final concentration of siRNA was 25 nM. Scrambled non-targeting siRNA (25 nM; Qiagen, Crawley,UK) was used as negative controls. Following 48 hour transfection supernatants were collected for analysis and cells lysed with TriReagent buffer (Sigma-Aldrich, Abingdon, UK) for total RNA extraction. Expression of NOD1 was determined as described below.

### Endothelial cell infection assay

Cells were untreated or inoculated with live *Haemophilus influenzae* (ATTC strain 49247), which is a Gram-negative bacterium. *Haemophilus influenzae* was used at colony forming unit dilutions of 10^8^–10^5^ for 24 hours. In these experiments a ‘filter control’ was also included. This control represents a bacteria free conditioned media produced by filtering cultures through a 30-kDa membrane (Ultrafree-0.5 PBTK Centrifugal Filter Unit 30 kDa Millipore UFV5BTK00) (Millipore, Gloucestershire, UK).

### Measurement of CXCL8

CXCL8 (IL8) was measured by ELISA (Duoset CXCL8 Kit, DY208E; R & D Systems, Abingdon UK), according to manufacturer's instructions.

### Measurement of cytokine array using MSD platform

The following cytokines were measured using the human pro-inflammatory 9-Plex MULTISPOT 96-well −10 spot MSD assay (Gaithersburg, Maryland, USA) (Cat no. N05007A-1); IL-2, IL-8, IL-12p70, IL-1β, GM-CSF, IFNγ, IL-6, IL-10 and TNFα. Selected samples from various experiments were diluted 1∶10 in Lonza-EGM2 and added to the MSD plate. The immunoassay was carried out according to manufacturer's instructions. Plates were read using an MSC Sector Imager 2400 and analysed using MSD Discovery Workbench software.

### Measurement of NF-κB translocation

Cells were treated for 1 hour with drugs as above, washed with PBS and fixed immediately in 4% para-formaldehyde (PFA) for 10 minutes at room temperature. Plates were then washed three times with PBS at 5 minute intervals. Plates were permeabilized with 0.2% Triton X-100 for 10 minutes and blocked with 4% foetal bovine serum (FBS) in PBS for 1 hour at room temperature. For NF-κB staining, cells were incubated with NF-κB-p65 (human) primary antibodies raised in rabbit (Santa Cruz Biotechnology, UK) for 1 hour at room temperature followed by secondary staining with AlexaFluor 546 anti-rabbit antibodies raised in goat (Invitrogen, UK) for 45 minutes at room temperature. Cells were washed three times between incubations with PBS at 5 minute intervals. Cell nuclei were stained with DAPI. Plates were then stored in PBS at −4°C prior to imaging using a Cellomics VTi HCS Arrayscanner (camera make/model: Arrayscan 12bit dynamic range high resolution thermo-cooled with a Zeiss Plan Neurofluour objective lens) (Thermo Fisher, Pittsburgh, USA). Some wells were treated with secondary antibody only to establish background auto fluorescence. Images were acquired at ×10 magnification at room temperature with PBS as imaging medium.

### *In vivo* conditioning of cells using transplantation in Matrigel

HESC-EC and HUVEC were expanded *in vitro* and 10^6^ cells were injected subcutaneously into 3-month-old athymic nude rats (Crl:NIH-*Foxn1^rnu^*, Charles River) in a suspension of 50 µl Matrigel (Becton Dickinson, Massachusetts, USA), heparin (64 U/ml), recombinant murine basic FGF (80 ng/ml, R & D Systems), 70 µl Lonza-EGM2. A matrigel suspension with no cells served as negative control. After 3 weeks, rats were sacrificed and plugs removed, photographed and stored for cryosectioning and RNA isolation. Animals used (n = 24) were RNU rats, Crl:NIH-Foxn1rnu, stain code 316. Anaesthetics ketamine (Richter Gedeon Pharmaceutical Company, Budapest, Hungary; 75 mg/kg, ip) and xylazine (Produlab Pharma, Rammsdonksveer, Netherlands; 5 mg/kg, ip) were used in surgical procedures.. Matrigel plugs with cells were lysed in TriReagent for total RNA extraction. The RNA was purified using RNeasy columns (Qiagen, Hilden, Germany), quantified, and checked for quality. 500 ng of total RNA was used for DNA generation using High Capacity cDNA Reverse Transcription Kit (Applied Biosystems, California, USA) according to manufactures instructions.

### Quantitative Real time PCR (qRT-PCR) for TLR4 and NOD1 expression

**Figure 1:**
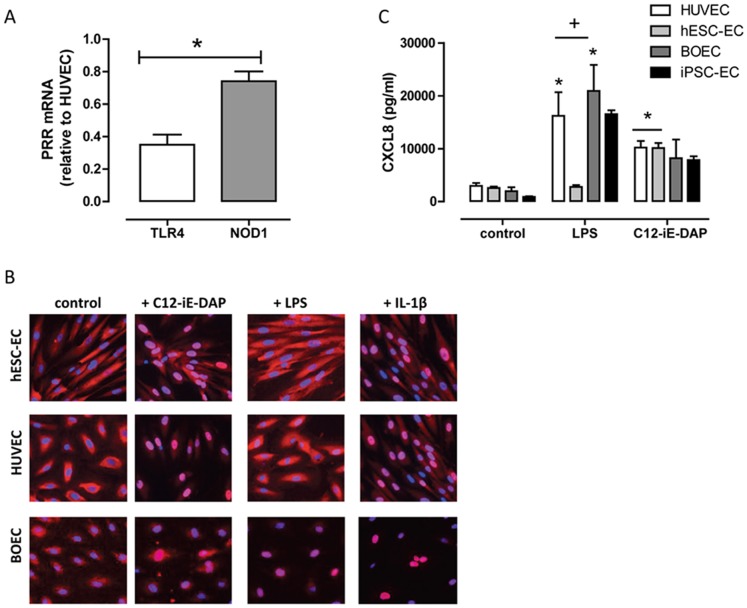
TLR4 and NOD1 expression and function in stem cell derived endothelial cells. (A) TLR4 and NOD1 expression in hESC-EC (relative to expression in HUVEC) *in vitro*. Data are mean ± SEM (n = 3). Statistical significance was determined by one-sample t-test (*p<0.05) for NOD1 vs. TLR4 expression. (B) Representative immunocytochemistry images of hESC-EC (top) and HUVEC (bottom) stained for the NF-κB p65-subunit (red) in response to 1 hour treatment with or without, C12-iE-DAP (NOD1 agonist; 10 µg/ml), LPS (TLR4 agonist; 1 µg/ml) or IL-1β (1 ng/ml). Nuclei were stained with DAPI (blue; 5 µg/ml). Images were acquired using a Cellomics VTi HCS Arrayscanner with a CarlZeiss microscope. (C) LPS (TLR4 agonist; 1 µg/ml) and C12-iE-DAP (NOD1 agonist; 10 µg/ml) induced CXCL8 release after 24 hour stimulation. Data are mean ± SEM. For HUVEC, hESC-EC or BOEC, n = 4–8. For iPSC-EC, n = 2, single isolation. Statistical significance was determined by one-way ANOVA followed by Dunnett's multiple comparison test for each cell type (*p<0.05) and by two-way ANOVA followed by Bonferroni's post-test for between cell types. Analysis was not performed on data from iPSC-EC.

For the PCR array (expression of TLR4 shown; Figure 1A) the cDNA was hybridized in a 96-well format against the Gene Array PAHS-058 with RT^2^ qPCR Master Mix, which contained SYBR green dye (RT^2^ Profiler PCR Array System, SABiosciences) as per the manufacturer's instructions. Data were normalised to the mean of 5 housekeeping genes included in the array (B2M, HPRT1, RPL13A, GAPDH, ACTB). Expression of NOD1 (shown in Figure 1A) was determined by qRT-PCR where RNA levels were determined using a NanoDrop platform and used to normalise loading of RNA prior to reverse transcription to cDNA for analysis. Data were normalised to GAPDH as a housekeeping geneThe PCR was performed with ABI 5700 (Applied Biosystems, CA) and Rotor-Gene 3000 (Corbett Research) real-time PCR instruments, and the relative expression was determined by ΔΔ^Ct^ method in which fold change = 2^−ΔΔCt^.

For quantifying mRNA levels of NOD1/CARD4 (Hs00196075_m1), and TLR4 (Hs00152939_m1) in hESC-EC pre- and post-implant and following NOD1-siRNA, real-time PCR analyses were performed with TaqMan Gene Expression Assays (Applied Biosystems, CA) using human-specific primers. GAPDH Endogenous Control (FAM/MGB probe) was used as a housekeeping control. Relative gene expression was determined by ΔΔ^Ct^ method in which fold increase = 2^−ΔΔCt^.

### Statistical analysis

All data is the mean ± S.E.M. for n separate incubations of individually treated cells. Unless otherwise stated all experiments were at least n = 3 and experiments were performed on at least 2 separate isolations of cells with separately prepared drugs or bacteria. Analysis was performed using GraphPad Prism software as described in each figure legend.

### Ethics Statement

Experiments using hESC and isolation of hESC-EC were approved by the UK Stem Cell bank. For the collection of human blood and the protocol for isolation of BOEC, ethical approval was granted by the Royal Brompton and Harefield Ethics Committee (ethics code: 08/H0708/69). Informed written consent was given by all participants. The consent procedure, and associated patient information sheets and consent forms, were approved by the Royal Brompton and Harefield Ethics Committee. Consent records were maintained as required by the Royal Brompton and Harefield Ethics Committee. For *in vivo* experiments using animals, the Animal Use and Care Committee of Semmelweis University Budapest approved the experimental protocols (Ref no. 22.1/1098/3/2011). The investigation conformed to the Guide for the Care and Use of Laboratory Animals published by the US National Institutes of Health.

## Results

### Cytokine, TLR4 and NOD1 agonist induced responses in hESC-EC, BOEC, iPSC-EC and HUVEC

We have previously shown that, whilst hESC-EC do not respond to TLR agonists (apart from TLR5) [11], they do express all of the necessary intracellular signalling to mount an immune/inflammatory response, and respond avidly to IL-1β [11]. Here we confirm our previous observations and show that hESC-EC expressed much lower levels of TLR4 than HUVEC (Figure 1A) and do not respond to LPS (Figure 1B and C). However, we show, for the first time, that hESC-EC do express a second PRR for Gram-negative bacteria, NOD1 (Figure 1A). In line with this, the NOD1 agonist C12-iE-DAP activated hESC-EC, causing nuclear translocation of NF-κB and release of CXCL8 (Figure 1B and C). Endothelial cells from umbilical veins (HUVEC) or derived from blood progenitors (BOEC) or from induced pluripotent stem cells (iPSC-EC) responded to both LPS and C12-iE-DAP (Figure 1C). In order to be sure that the lack of response to LPS seen at the level of CXCL8 was not specific to this chemokine, we measured release of a range of other cytokines (GM-CSF, IFNγ, IL-10, IL-12p70, IL-1β, IL-2, IL-6, TNFα), and found identical responses (Table 1 and 2). The NOD1 agonist, C12-iE-DAP induced release of GM-CSF, IL-12q70, IL-2, IL-6, IL1β and TNFα from hESC-EC with no effect seen with LPS (Table 1 and 2).

**Table 1:** MSD analysis of cytokine (pg/ml) release from hESC-EC.

Analyte (pg/ml)	GM-CSF	IFNγ	IL-10	IL-12p70	IL-1β	IL-2	IL-6	TNFα	CXCL8
CONTROL	30.3±2.5	ND	1.6±0.4	8.0±3.6	0.8±0.3	65.9±5.9	90.9±14.5	ND	1566.5±160.0
+ C12-iE-DAP	160.9±45.3*	11.1±7.1	6.0±1.7	83.7±30.3*	11.9±1.1*	303.9±64.8*	295.5±78.1*	4.8±0.2*	9569.1±2645.2*
+ LPS	20.9±0.5	ND	1.9±0.3	12.9±3.2	2.1±1.2	62.6±4.0	104.8±9.1	ND	1691.8±120.3

Data mean are ± SEM for n = 3. hESC-EC were treated for 24 hours with vehicle, LPS (1 µg/ml), or C12-iE-DAP (10 µg/ml). Statistical significance was determined by one-way ANOVA followed by Dunnett's multiple comparison test (*p<0.05). ND = non-detectable.

**Table 2:** MSD analysis of cytokine (pg/ml) release from HUVEC.

Analyte (pg/ml)	GM-CSF	IFNγ	IL-10	IL-12p70	IL-1β	IL-2	IL-6	TNFα	CXCL8
CONTROL	37.0±1.6	2.8±1.0	1.4±1.2	12.9±0.9	1.3±0.6	69.3±19.2	63.7±20.4	0.5±0.3	1500.6±351.5
+ C12-iE-DAP	190.0±44.8	7.9±3.0	2.7±1.1	60.2±12.5*	7.0±3.3	198.2±26.3*	213.1±65.1	1.7±0.9	6044.1±960.3*
+ LPS	279.±115.5	5.1±1.3	4.7±0.6	38.7±5.0	5.6±2.4	223.1±34.3*	323.8±114.2	1.8±1.0	6154.4±857.6*

Data are mean ± SEM for n = 3. HUVEC were treated for 24 hours with vehicle, LPS (1 µg/ml), or C12-iE-DAP (10 µg/ml). Statistical significance was determined by one-way ANOVA followed by Dunnett's multiple comparison test (*p<0.05).

### Effect of *in vivo* conditioning of hESC-EC following implantation into nude rats

**Figure 2:**
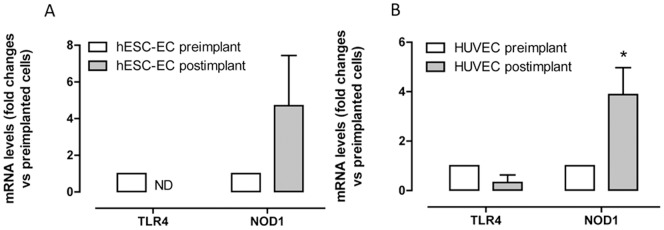
Effect of *in vivo* ‘conditioning’ on TLR4 and NOD1 expression. TLR4 and NOD1 expression in (A) hESC-EC and (B) HUVEC before (pre-implant; open bars) and 21 days after (post-implant; filled bars) implantation *in vivo* (‘conditioning’). Data are mean ± SEM and are normalized at unity (1) to gene levels in pre-implant cells. HUVEC; NOD1 pre-implant n = 8, post implant n = 4: HUVEC; TLR4 pre-implant n = 10, post implant n = 3. hESC-ECs; NOD1 pre-implant n = 6, post implant n = 5: hESC-ECs; TLR4 pre-implant n = 10, post implant n = 6. Data was obtained from 2 independent experiments (using up to 12 rats per group). Statistical significance was determined by one-sample t-test where results were compared to a theoretical control of 1 (*p<0.05). ND = none detectable.

The hESC-EC used here were differentiated into endothelial cells using standard protocols and, as we have shown previously, display hallmarks of mature endothelial cells [11]. However, in order to establish if TLR4 expression could be induced *in vivo*, cells were transplanted into nude rats and ‘conditioned’ for 21 days. This approach has been shown by others to result in vessel formation of hESC-EC *in vivo* [21], [22]. However, after transplant *in vivo*, TLR4 was not increased in hESC-EC or in HUVEC (Figure 2). By contrast, NOD1 expression tended to be increased in both hESC-EC and HUVEC after *in vivo* conditioning. These observations suggest that hESC-EC will retain an immune privileged phenotype for TLR4 and an active NOD1 pathway when transplanted *in vivo*.

### Responses of hESC-EC and HUVEC to *Haemophilus influenzae* infection

**Figure 3:**
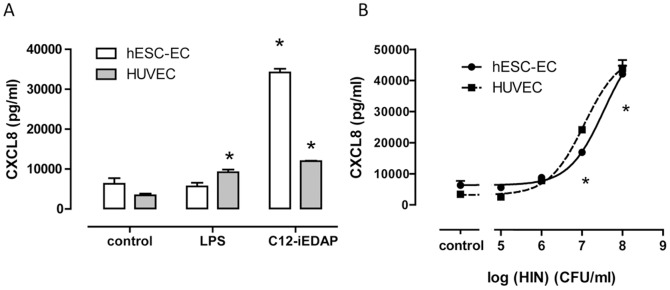
Responses of hESC-EC and HUVEC to 24 hour infection with *Heamophilus influenzae.* (A) Effect of LPS (1 µg/ml) or C12-iE-DAP (10 µg/ml) on CXCL8 release from hESC-EC and HUVEC after 24 hours. (B) Effect of *Haemophilus influenzae* (HIN) (10^5^–10^8^ CFU/ml) on CXCL8 release from hESC-EC (solid line) or HUVEC (dashed line) after 24 hours. Data are mean ± SEM; n = 3 representative of 6 hESC-EC isolations. Statistical significance for responses to drugs or bacteria was determined by one-way ANOVA followed by Dunnett's multiple comparison test (p<0.05).

As discussed, Gram-negative bacteria are sensed by two key PRR pathways: TLR4, which is the receptor for LPS, and NOD1, which is the receptor for peptidoglycan moieties. As with other types of Gram-negative bacteria, *Haemophilus influenzae* is reportedly sensed by cells via TLR4 and/or NOD1 PRR pathways [23], [24]. However, the relative contribution of TLR4 versus NOD1 to sensing of whole live Gram-negative bacteria will vary depending upon the cell type. In order to test the potential for hESC-EC to sense bacteria, despite no TLR4, we infected hESC-EC and HUVEC with live Gram-negative bacteria (*Haemophilus influenzae*). In these experiments, as with others, basal release of CXCL8 was relatively low in both cell types and again, LPS activated HUVEC, but not hESC-EC, whilst C12-iE-DAP activated both cell types (Figure 3A). However, despite no TLR4 function, infection of hESC-EC with live *Haemophilus influenzae* induced a concentration-dependent release of CXCL8 (Figure 3B). Importantly, the potency and efficacy of *Haemophilus influenzae* to induce CXCL8 release was found to be comparable between hESC-EC and HUVEC (Figure 3). These results suggest that, in hESC-EC, NOD1 receptors are sufficient to accommodate the sensing of Gram-negative bacteria. In order to establish the role of NOD1 receptors in the activation of hESC-EC by *Haemophilus influenzae*, we took a molecular approach using gene knock down, and a pharmacological approach using selective inhibitors.

### Role of NOD1 in hESC-EC responses to C12-iE-DAP and Gram-negative bacteria

**Figure 4:**
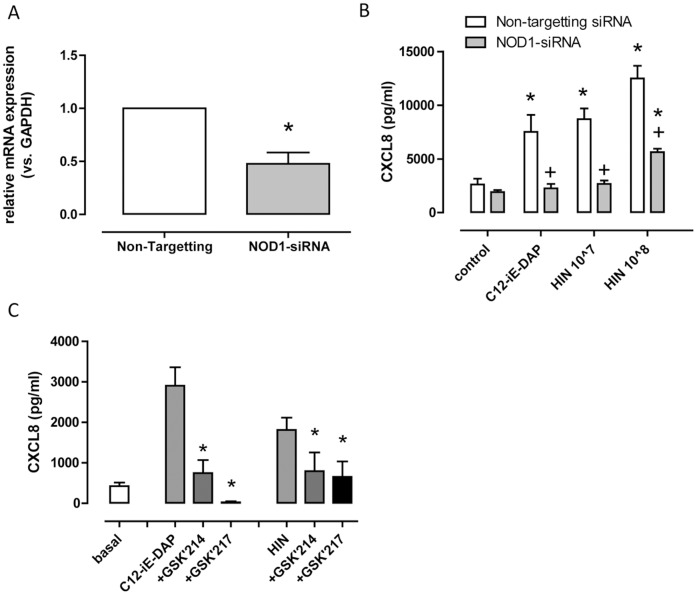
Effect of pharmacological inhibition of RIP2 and NOD1 siRNA mediated knockdown on responses of hESC-EC to *Haemophilus influenzae* (HIN) and C12-iE-DAP. (A) Relative expression (vs. GAPDH) of NOD1 following 48 hour incubation with NOD1 siRNA normalized to non-targeting siRNA; n = 6. (B) CXCL8 release from hESC-EC following 48 hour pre-incubation with non-targeting siRNA (open bars) or NOD1-siRNA (filled bars) and 24 hour treatment +/− C12-iE-DAP (10 µg/ml) or *Haemophilus influenzae (HIN)* (10^7^–10^8^ CFU/ml); n = 7–8. (C) Effect of GSK'214 (300 nM; RIP2 inhibitor) or GSK'217 (300 nM; NOD1 inhibitor), given 30 minutes before a 24 hour treatment with HIN (10^7^ CFU/ml) or C12-iE-DAP (10 µg/ml) on CXCL8 release; n = 4. It should be noted that GSK drugs increased CXCL8 release under basal conditions; for each experiment this was subtracted from treatment groups. For panel A, statistical significance was determined by one-sample t-test. For panel B statistical significance within siRNA groups was determined by one-way ANOVA followed by Dunnett's multiple comparison test (*p<0.05), and between groups by two-way ANOVA followed by Bonferroni's post-test (+p<0.05). For panel C statistical significance for the effects of inhibitor of C12-iE-DAP or HIN induced CXCL8 was determined by one-way ANOVA followed by Dunnett's multiple comparison test (*p<0.05).

NOD1 receptors can be knocked down using conventional targeting siRNA. In addition, novel NOD1 and RIP2 inhibitors have been developed by GSK, which we have previously used and validated [25]. We have shown that the NOD1 antagonist GSK'217 inhibits NOD1, without affecting TLR4 in endothelial cells [25], and that the RIP2 inhibitor GSK'214 also blocks NOD1 without affecting TLR4 responses in these cells [25]. Here we show, as expected, that knocking down NOD1 receptors at the gene level (Figure 4A) inhibited responses in hESC-EC to the NOD1 agonist C12-iE-DAP and, importantly, also to *Haemophilus influenzae* infection (Figure 4B). In line with this, inhibiting NOD1 with GSK'217 or RIP2 with GSK'214 reduced CXCL8 release from *Haemophilus influenzae* infected or C12-iE-DAP treated cells (Figure 4C). CXCL8 levels from IL-1β treated hESC-EC were not affected by NOD1 siRNA (Figure S1) or the NOD1 (GSK'217) and RIP2 (GSK'214) inhibitors (Figure S2).

## Discussion

Endothelial cells derived from stem cells have a plethora of potential applications in pharmacology and regenerative medicine. In the short term, the most important feature of any regenerated vasculature must be a resistance to thrombosis. Without this property, the graft would fail, as thrombi and clots prevent adequate perfusion. However, in the longer term any vascular graft needs to be resistant to atherosclerosis and restenosis.

The link between atherosclerosis and inflammation has been known for some time. In 1988, a limited number of studies made correlations between markers of bacterial infection and coronary artery disease in man [26]. In line with these studies, others have shown that Gram-negative Chlamydia is present in atherosclerotic lesions [27]. These papers paved the way for early clinical trials designed to assess the potential preventive benefits of antibiotic therapy in atherosclerosis [28]. Whilst, on the whole, the clinical trials with antibiotics were not successful in preventing cardiovascular disease, the link between pathogens and atherosclerosis has continued to be investigated. We now know the innate immune receptors, TLR4 and TLR2, intrinsically regulate atherosclerosis in animal models [7] and induce inflammatory responses in human vascular cells [25], [29].

We have previously shown that endothelial cells derived from human stem cells (hESC-EC), have no functional TLR4 or TLR2 and, whilst it is beyond our current ability to test, we have speculated that this may afford these cells an athero-protected privilege over other endothelial cells derived from other stem cell sources [10], [11]. In the current study, we have confirmed our previous observation that hESC-EC have no TLR4 response, but that they have a fully functioning MyD88 pathway since they respond avidly to IL-1β. We have gone on to perform a unique comparison of the TLR4 response profile in endothelial cells derived from the key stem cell sources, namely embryonic stem cells, blood progenitor cells and induced pluripotent stem cells.

As with endothelial cells derived from adult [11], [25] or foetal vessels (HUVEC; this paper), but in direct contrast to endothelial cells from embryonic stem cells (hESC-EC), we found the endothelial cells derived from blood progenitors (BOEC), or induced pluripotent stem cells (iPSC-EC), responded avidly to LPS. These observations support the idea that hESC-EC may be athero-protected, if remaining TLR4 resistant *in vivo*.

It is beyond the scope of this study or current technology to investigate the long term stability of transplanted hESC-EC in regard to atherosclerosis. However, we have used the technology that is available currently and performed experiments to determine the effect of the *in vivo* environment on TLR4 expression. This type of *in vivo* conditioning has been shown to confer maturation of part-differentiated stem cells to fully mature erythroid [30] or pancreatic islet cells [31]. As mentioned above, in comparison with HUVEC, we found that hESC-EC had very low TLR4 gene expression but relatively high NOD1. Similarly to previous approaches [21], [22], endothelial cells were injected into Matrigel plugs implanted beneath the skin of nude rats. Nude rats have compromised immune responses and so do not ‘reject’ the human cell transplant. In our model, after the endothelial cells had been incubated *in vivo*, we specifically measured human TLR4 and NOD1 gene expression. This allowed us to differentiate PRR expression in the human cells from those of the host (rat) cells. Following *in vivo* conditioning, TLR4 expression remained low/absent in hESC-EC. By contrast, NOD1 levels were stable in both hESC-EC and HUVEC *in vivo*. Whilst not definitive, these experiments are consistent with the idea that hESC-EC will retain their TLR4-deficient phenotype *in vivo*, and supports our hypothesis that, through this property, they would be resistant to atherosclerosis.

NOD1 expression in hESC-EC was accompanied by a fully functional cellular response to NOD1 agonists. Specifically, we found that the NOD1 agonist C12-iE-DAP activated NF-κB and induced cytokine release by hESC-EC. Importantly, the relative sensitivity of hESC-EC to NOD1 agonists was similar to that seen in endothelial cells derived from our other stem cell sources and in HUVEC.

Whilst we speculate that absence of TLR function may result in protection from atherosclerosis, it is important to recognise that functionally, endothelial cells should be able to sense pathogens and mount an immune response. If endothelial cells are totally insensitive to bacteria this may render a vessel or organ ‘immunosuppressed’. This is important to consider in cell/organ therapy as post-organ transplant infection represents a major clinical problem [32]–[34]. Our findings that hESC-EC could sense NOD1 agonists, despite having no TLR4 function, led us to consider that these cells had the potential to sense bacteria via the NOD1 pathway. To test this we infected cells with *Haemophilus influenzae* Gram-negative bacteria and found that despite no TLR4, hESC-EC mounted a robust inflammatory response. This was reduced in cells where NOD1 had been knocked down using targeting siRNA, or by selective inhibition of NOD1 with a prototype NOD1 inhibitor (GSK217; [25]), or inhibition of RIP2, the obligatory signalling pathway for NOD receptors. This indicates that, despite lack of TLR4 activation, hESC-EC are capable of responding to Gram-negative bacteria via NOD1 pathways, and so would be functional to mount a defence when incorporated into a graft.

In summary, we show that hESC-EC are unique amongst stem cell-derived endothelial cells since they do not express functional TLR4, even after *in vivo* conditioning. We show that, despite the lack of TLR4, these cells can sense Gram-negative bacteria via a fully functional NOD1 pathway. We speculate that endothelial cells lacking TLR4 may be protected from atherosclerosis. It must be noted however, that the role of NOD1 in atherosclerosis is not yet known. Thus, whilst our results clearly show that TLR4 remains a deficient pathway in endothelial cells from embryonic stem cells, our hypothesis should be viewed with caution until we know more about (i) the fate of these cells in vivo in a disease setting and (ii) NOD1 in vascular inflammation. Finally, it should also be noted that the embryonic stem cell might not represent the best stem cell progenitor in every therapeutic scenario especially given their potential for allogenecity. We should then also consider the potential to engineer ‘TLR4-deficient’ endothelial cells from host stem cells such as those found in blood.

The initial data included here, showing more complete TLR4 responses in blood- and iPSC-derived endothelial cells is interesting, and future experiments should aim to confirm whether this is a systematic difference between hESC, iPSC and adult stem cells. The idea of modifying stem cell-endothelial cells to improve therapeutic utility is not new [10], [35]. Thus, characterisation and modification of stem cell-endothelial cells at the level of TLR4 and other pattern recognition receptors represents a potentially important target for optimal and tailored cell therapy design.

## Supporting Information

Figure S1 **Effect of NOD1 siRNA targeting on IL-1β induced CXCL8 release.** CXCL8 release from hESC-EC following 48 hour pre-incubation with non-targeting siRNA (open bars) or NOD1-siRNA (filled bars) and 24 hour treatment with/without IL-1β (0.01–0.1 ng/ml). Data are mean ± SEM (n = 6–8).
(TIF)

Figure S2 **Effect of GSK'214 and GSK'217 on IL-1β induced CXCL8 release.** CXCL8 release from hESC-EC following 30 minute pre-incubation with GSK'214 (300 nM) or GSK'217 (300 nM) and 24 hour treatment with/without IL-1β (0.1 ng/ml). Data are mean ± SEM (n = 4). Data were handled as in Figure 4D.
(TIF)
