# Supplementary figures and images for: Pathogen Sensing Pathways in Human Embryonic Stem Cell Derived-Endothelial Cells: Role of NOD1 Receptors
Source: PLoS One. 2014 Apr 1;9(4):e91119. doi: 10.1371/journal.pone.0091119 (PMC3972153; doi:10.1371/journal.pone.0091119)

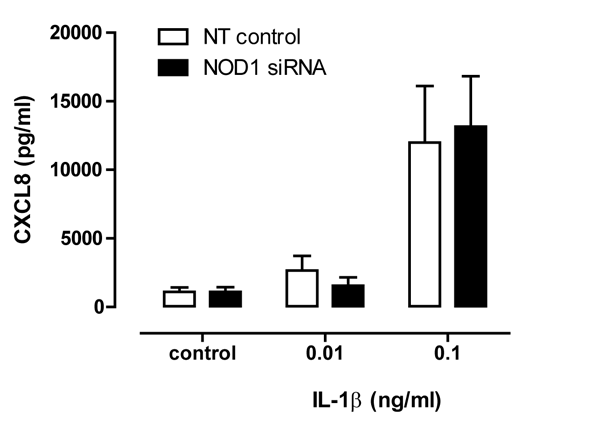

Supplement: Figure S1 — Effect of NOD1 siRNA targeting on IL-1β induced CXCL8 release. CXCL8 release from hESC-EC following 48 hour pre-incubation with non-targeting siRNA (open bars) or NOD1-siRNA (filled bars) and 24 hour treatment with/without IL-1β (0.01–0.1 ng/ml). Data are mean ± SEM (n = 6–8). (TIF) [file pone.0091119.s001.tif]

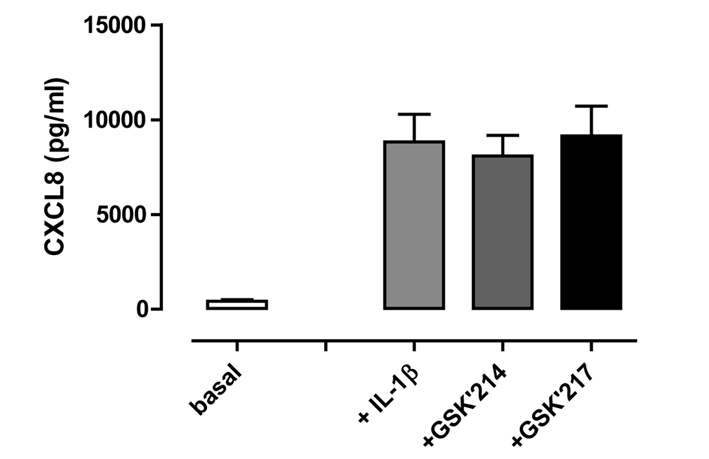

Supplement: Figure S2 — Effect of GSK'214 and GSK'217 on IL-1β induced CXCL8 release. CXCL8 release from hESC-EC following 30 minute pre-incubation with GSK'214 (300 nM) or GSK'217 (300 nM) and 24 hour treatment with/without IL-1β (0.1 ng/ml). Data are mean ± SEM (n = 4). Data were handled as in Figure 4D. (TIF) [file pone.0091119.s002.tif]
